# Human Papillomavirus 16 DNA Methylation Patterns and Investigation of Integration Status in Head and Neck Cancer Cases

**DOI:** 10.3390/ijms241914593

**Published:** 2023-09-26

**Authors:** Ioannis Zygouras, Danai Leventakou, Abraham Pouliakis, Styliana Panagiotou, Dimitris Tsakogiannis, Georgios Konstantopoulos, Eirini Logotheti, Menelaos Samaras, Zaharoula Kyriakopoulou, Apostolos Beloukas, Ioannis S. Pateras, Alexandros Delides, Amanda Psyrri, Ioannis G. Panayiotides, Minas Yiangou, Christine Kottaridi

**Affiliations:** 1 Department of Genetics, Development and Molecular Biology, School of Biology, Aristotle University of Thessaloniki, 54124 Thessaloniki, Greece; izygouras@bio.auth.gr (I.Z.); stylianap@bio.auth.gr (S.P.); konstanto@bio.auth.gr (G.K.); eirinilg@bio.auth.gr (E.L.); yiangou@bio.auth.gr (M.Y.); 2 2nd Department of Pathology, University General Hospital “Attikon”, School of Medicine, National and Kapodistrian University of Athens, 12464 Athens, Greece; dleventakou@med.uoa.gr (D.L.); apouliak@med.uoa.gr (A.P.); menelaos.g.samaras@gmail.com (M.S.); ispasath2004@yahoo.com (I.S.P.); ioagpan@med.uoa.gr (I.G.P.); 3 Research Center, Hellenic Anticancer Institute, 10680 Athens, Greece; 4 Department of Environment, School of Technology, University of Thessaly, Gaiopolis Campus, 41500 Larissa, Greece; zahkyr@uth.gr; 5 Department of Biomedical Sciences, University of West Attica, 12243 Athens, Greece; abeloukas@uniwa.gr; 6 National AIDS Reference Centre of Southern Greece, School of Public Health, University of West Attica, 11521 Athens, Greece; 7 2nd Department of Otolaryngology, University General Hospital “Attikon”, School of Medicine, National and Kapodistrian University of Athens, 12464 Athens, Greece; adelidis@med.uoa.gr; 8 2nd Department of Internal Medicine-Propaedeutic, University General Hospital “Attikon”, School of Medicine, National and Kapodistrian University of Athens, 12464 Athens, Greece; psyrri237@yahoo.com

**Keywords:** HPV16, L1, UTR methylation, viral integration, HNSCC

## Abstract

Persistent high-risk human papillomavirus (HPV) infection is a pivotal factor in the progression of cervical cancer. In recent years, an increasing interest has emerged in comprehending the influence of HPV on head and neck squamous cell carcinoma (HNSCC). Notably, it is well established that HPV-associated HNSCC show cases with distinct molecular and clinical attributes compared to HPV-negative cases. The present study delves into the epigenetic landscape of HPV16, specifically its *L1* gene and untranslated region (UTR), through pyrosequencing, while the HPV16 DNA physical status was evaluated using E2/E6 ratio analysis in HPV16-positive HNSCC FFPE biopsies. Our findings reveal substantial methylation across six sites within the HPV16 *L1* gene and seven sites in the UTR. Specifically, methylation percentages of two L1 CpG sites (7136, 7145) exhibit significant associations with tumor histological grade (*p* < 0.01), while proving concurrent methylation across multiple sites. The HPV16 DNA physical status was not correlated with the methylation of viral genome or tumor characteristics. This is the first study that examines epigenetic modifications and the HPV16 DNA physical status in Greek HNSCC patients. Our findings suggest an orchestrated epigenetic modulation among specific sites, impacting viral gene expression and intricate virus–host interactions.

## 1. Introduction

Head and neck cancers are a broad group of tumors that can be found in the head and neck area, such as the oropharynx, nasopharynx, larynx, oral cavity and hypopharynx [1]. They are the seventh most common cancer worldwide and in 2016 there were more than 1.1 million new cases of head and neck cancers reported all over the world, which eventually caused about 500,000 deaths [2]. Alcohol and tobacco significantly increase the risk of developing head and neck cancer but, at present, there is a steady increase in reporting the HPV-related cancers that most frequently affect the oropharynx. The reason why HPV shows a preference for the oropharynx is not yet clear; it could be related, according to studies, to the presence of transitional mucosa which bears histological similarities to the cervical mucosa [3,4].

Human papillomavirus (HPV) belongs to the Papillomaviridae (PV) family and is known for its ability to infect the oral and genital mucosal epithelium and skin [5]. It is a circular double-stranded DNA virus of small size, and its genome is encapsulated in icosahedral capsids consisting of roughly 8000 bp without an envelope [6]. Three regions can be distinguished: the early (E), that manages replication (E1, E2, E4, E5, E6, E7), the late (L), that encodes the major and minor capsid proteins (L1, L2) and the long control region (LCR) or non-coding region (NCR) or upstream regulatory region (URR) or untranslated region (UTR) which is located downstream of the *L1* gene and before the *E6* gene and regulates DNA replication by controlling the transcription of viral genes [6,7].

To date, based on the NCBI database, there are around 500 different PV types, while over 220 of them can infect humans, with 40 being associated with malignancies in the anogenital tract as well as in the head and neck region (PaVE: The Papillomavirus Episteme; Available online: https://pave.niaid.nih.gov/index, accessed on 12 June 2023) [8]. They can further be grouped as high-risk (HR) or low-risk (LR) genotypes, with HR types considered to be highly oncogenic and able to cause premalignant and malignant lesions [8,9].

The oncogenes *E6* and *E7* found in high-risk HPV types interfere with cell cycle regulation by affecting tumor suppressor genes [10]. E6 protein targets p53, binds with it and degrades it, thus allowing damaged or mutated cells to continue through the cell cycle, leading to the accumulation of mutations [6]. E7 protein, on the other hand, binds to retinoblastoma protein (Rb) and prevents its interaction with the E2F transcription factor, resulting in the activation of E2F and progression of the cell cycle to the synthesis phase [6].

As it is known, oncoproteins of HR-HPV types affect the expression of host genes that are associated with epigenetic changes and especially those that affect the methylation of either viral or host DNA [11]. Methylation involves the addition of a methyl group to the 5′ carbon of the pyrimidine ring of cytosine in CG dinucleotides. Typically, gene expression is controlled by cytosine methylation, which affects regulatory sequences like promoters, leading to the genes’ silencing [12,13]. In cancers, disruptions to DNA methylation patterns are often observed and, in some cases, these changes may inactivate tumor suppressor genes and activate oncogenes [14].

Persistent infection with HR-HPV types can lead to the integration of the viral genome into the chromosomes of the host, thus causing cancer [15]. Viral DNA is circular in its episomal form, but it becomes linear when it integrates into the host genome [16]. Integration of the virus requires the breakage of the viral and host DNA. Usually, integration affects the *E2* gene, leading to its deletion or truncation and causing a loss of E2 protein production, which normally works as a suppressor for *E6* and *E7* oncogenes [17]. Thus, those genes are expressed uncontrollably and biological functions, like cell death and proliferation, two key cancer hallmarks, are dysregulated [17].

Despite the large number of studies covering the methylation and integration of HPV16 virus in cervical cancer, there remains a scarcity of investigations concerning epigenetic alterations of HPV16 in head and neck cancers. Patients with high methylation levels at E2 binding sites (E2BS3 and E2BS4), which are located in the UTR, displayed the highest E6 and E7 expression levels and tended to have worse 5-year overall survival compared with patients with intermediate methylation levels [18]. HPV *L1* methylation has also been detected in oral squamous cell carcinomas, and elevated levels of methylation of HPV 16 late genes may be useful in predicting or detecting oral HPV infections at risk of progression to oropharyngeal cancer [19]. Most studies have focused on assessing the integration of viral DNA in HNSCC cases by analyzing the E2/E6 ratio, revealing varying frequencies of viral integration events.

Compared to cervical cancer, research has indicated that the level of methylation in UTR is associated with the physical state of the virus, whether it exists in an integrated or episomal form. Some studies have found significantly or moderately increased methylation in this region, while others report low levels of methylation in the HPV16 UTR. In contrast, investigations into the methylation patterns of the HPV16 *L1* gene consistently reveal a high level of methylation.

The present study aims to investigate the interplay between viral presence and the host milieu within this disease context. We concentrate on assessing the methylation status of the *L1* gene and untranslated region (UTR) in a small cohort of clinical samples. Methylation patterns within the CpG sites of the HPV16 *L1* gene were investigated due to the pertinent literature on cervical cancer, which hints at specific positions in the *L1* gene as potential indicators of disease progression. Our focus on the *L1* gene aimed to address whether these particular *L1* sites are impacted in HNSCC and which their correlation might be with histological grade. Our objective was also to identify E2 binding sites belonging to the p97 promoter region. More specifically, UTR37 and UTR43 are located in the E2BS3 (E2 Binding Site 3), while UTR52 and UTR58 are located into the E2BS4 (E2 Binding Site 4). The methylation of CpG dinucleotides within the E2 binding sites (E2BSs) in the HPV16 UTR can modify the binding affinity of the E2 protein. This, in turn, leads to the activation of the p97 promoter and subsequently enhances the transcription of *E6* and *E7* in the presence of E2.

Through this endeavor, we aim to illuminate the intricate dynamics between HPV16 and the host genome, shedding light on the potential associations with cancer progression and disease severity.

## 2. Results

Clinical samples from 31 men aged 38–82 years (median = 58) at the time of diagnosis and 7 women aged 48–69 years (median = 54) at the time of diagnosis were studied. Most studied specimens were derived from the larynx (*n* = 17, 44.74%), while the tonsils was the next anatomic position with increased number of samples among the investigated material. The real time PCR diagnostic test for the genotyping of Human Papillomavirus types (16, 18, 31, 33, 35, 39, 45, 51, 52, 56, 58, 59, 66 and 68) resulted in the detection of HPV16 exclusively. Correlations between HPV16 viral genes methylation and the cancer grade, as well as between HPV16 integration and cancer grade, were investigated.

**Figure 1:**
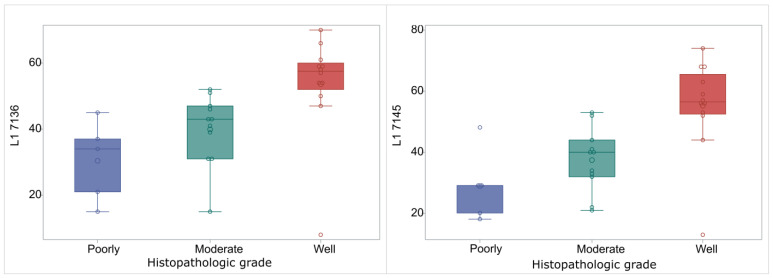
Box and whisker plot for L1 7136 and L1 7145 expression in relation to tumor grade. Box limits show Q1 and Q3 values. The lines within the boxes correspond to median values, the large circles indicate the median values and smaller circles correspond to measurements. Outliers are the circles outside the whisker limits, (**left**): L1 7136, (**right**): L1 7145.

A reliable pyrosequencing assay was used after the bisulfite conversion of DNAs and the quantification of methylation percentage in different CpG sites of UTR, and *L1* gene of HPV16, was assessed. The mean methylation of 5′ UTR sites ranged from 9.71 ± 13.85 to 32.55 ± 23.03 while, for *L1* gene sites, the pyrosequencing analysis revealed that the methylation percentages were 37.11 ± 24.43 to 43.84 ± 16.69 (Table 1). The median methylation percentage and the Q1–Q3 range for each CpG site for the different histological grades were calculated and are depicted at Table 2. As shown, a statistically significant correlation of methylation percentage to histological grade was obvious for the sites L1 7136 and L1 7145. There is a clear correlation of these two L1 CpG sites with the tumor grade and specifically the methylation percentages are higher in cases of histologically well-differentiated HNSCC cases (Figure 1).

Correlations between all possible methylation sites are presented in detail in Table 3; there are 78 possible pairs of the studied sites, statistically significant positive correlations (*p* < 0.05 or even lower, see Table 3) were found in 49, thus it is clear that methylation is a phenomenon that occurs simultaneous in many sites. UTR7270s and L1-7034 were the two sites at which significant correlations were not found with the other methylation sites, in the other sites (a) UTR31, UTR37, UTR43, UTR52 and UTR58 were all very strongly correlated with correlation coefficients more than 90% (*p* < 0.0001) and, (b) for the sites UTR_7862, UTR_7270, L1_7034, L1_7091, L1_7136, L1_7145, L1_6367 and L1_6457, there were strong or medium (correlation coefficients <50% and <70%, respectively) or lower, indicative for low or no correlation.

The HPV16 DNA physical status was evaluated using the E2/E6 ratio in the examined samples. According to our results, the pure episomal HPV16 DNA form was detected in 12 out of 38 samples (31.6%). Moreover, the mixed DNA form was identified in 16 out of 38 cases (42.1%), while the pure integrated HPV16 DNA form was found in 10 out of 38 studied cases (26.3%). Subsequently, the results derived from E2/E6 ratio analysis were further associated with the histological grade, the tumor anatomic position and the methylation percentage of different CpG sites of UTR and the *L1* gene of HPV16, respectively. According to our outcomes, no statistically significant associations were recorded. Nevertheless, more analyses of larger sample sizes are required to assess whether HPV16 DNA physical status is associated with tumor characteristics and the methylation of the viral genome.

## 3. Discussion

An increasing number of head and neck squamous cell carcinoma (HNSCC) at-tributed to the high-risk oncogenic HPV types is recorded. HPV16 type is reported as being responsible for around 70% in the USA and 52% in the UK of all oropharyngeal squamous cell carcinoma (OPSCCs) [20,21]. Around 99% of HPV infections are caused by HR types 16, 18, 31 and 33, with HPV16 being the type that is most common [22,23].

The major risk factors for HNSCC have a well-established predominant link to smoking and heavy alcohol use and are usually HPV negative. HPV(+) and HPV(−) head and neck cancers have different characteristics concerning their epidemiology, biology and treatment, so they are considered as different entities [24]. High-risk human papillomaviruses (HPV), and in particular HPV16, are most strongly associated with oropharyngeal squamous cell carcinomas (OPSCC). Patients with HPV(+) HNSCC have a higher positive treatment response and survival than those individuals with HPV(−) HNSCC. Patients with HPV(+) HNSCC respond better to radiation and live longer [25]. Although HPV(+) and HPV(−) head and neck cancers have similar overall mutation rates and mutational burdens [26], HPV(+) tumors exhibit much more aberrant DNA methylation patterns than HPV(−) head and neck tumors [27,28].

Amidst the extensive research conducted on the methylation and integration of the HPV16 virus in cervical cancer, the current knowledge regarding head and neck cancers remains scarce. Therefore, the primary objective of this study was to better understand the relationship between viral presence and the host. This investigation focused on examining the methylation patterns of the *L1* gene and the UTR region as well as the physical status of the virus itself within a small cohort from the Greek population. Through this study, we anticipated gaining valuable insights into the host–virus interactions and, therefore, the potential impact on cancer advancement and the severity of the disease, paving the way for suggesting personalized biomarkers that promise improved patient outcomes.

According to our results, we observed that the *L1* region is highly methylated with mean values ranging from 37.11 ± 24.43 to 43.84 ± 16.69 at the studied CpG sites, as previously observed at the same sites in cervical cancer tissues [29]. It is clear that the viral E6 and E7 oncoproteins interact with the host’s DNA methyltransferase machinery, in a similar manner to in different anatomical locations, driving the high methylation percentages to viral *L1* gene.

The HPV16 UTR is crucial for controlling the expression of the viral genes. The 3′ UTR-located P97 promoter, where the studied 7862, 31, 37, 43, 52, 58 sites are located, regulates the transcription of the HPV16 *E6* and *E7* oncogenes through a feedback mechanism, which is controlled by the viral E2 protein. Studies on HPV16 in cervical cancer have shown that the degree of methylation in this region, which is either significantly or moderately elevated, is related to the physical status of the virus, which is relevant to its integrated or episomal form [30,31,32], while other studies report that the mean methylation of HPV16 UTR shows constantly low methylation percentages [33]. In particular, oncogene transcription is thought to be restricted if E2 viral protein is unable to bind at certain sites because of inhibition by methylated cytosines inside its binding site, indicating that the episomal physical status of the virus is probably present in such cancerous cases. Regarding the UTR, in the present study, the mean methylation values ranged from 9.71 ± 13.85 to 32.55 ± 23.03, with the sites located at the p97 promoter having the highest methylation percentages.

One of the most important findings of this study is the significant correlation of the sites L1 7136 and L1 7145 with the histological grade of this disease. The pyrosequencing assay resulted in higher methylation percentages in clinical samples with well-differentiated tumors, while obvious lower methylation percentages at the same sites were depicted into poorly or even moderately differentiated samples. Such an investigation of the relationship between methylation and histological grade may provide valuable insights into the underlying molecular mechanisms leading to disease progression in the presence of HPV. A possible scenario, that undoubtedly needs further investigation, suggests that the high *L1* gene levels of methylation into the well-differentiated histologically cases is related to the mechanistic pathways involved in cell differentiation and regulation that correlate with the high activity of DNA methyltransferase. On the other hand, the lower methylation levels of poorly and moderately differentiated cancer may be associated with gene regulatory pathways, leading to more aggressive forms of carcinomas at the specific anatomical site. To the best of our knowledge, our study is the first one to show the association between the methylation of *L1* HPV16 gene and the differentiation status in head and neck cancer cases originated from Greece.

Notably, our study demonstrated strong correlations between different UTR sites, indicating that methylation occurs simultaneously in these regions, suggesting that the underlying epigenetic mechanism acts in an orchestrated manner among specific sites intending to influence viral gene expression and the interaction with the host.

The integration of HPV16 DNA into the host chromosome is regarded as a crucial event during malignant transformation and cancer progression [16]. The detection of integrated viral DNA in cervical samples seems to be a considerable molecular tool that enables the prediction of cervical cancer development [34]. However, it remains unclear whether HPV16 DNA integration augments patients’ vulnerability to the development of oropharyngeal cancer. At present, only limited data concerning the physical status of HPV16 DNA in the development of oropharyngeal malignancy are available. In particular, previous findings revealed the high frequency of the mixed HPV16 DNA form in 18 fresh biopsies from oropharyngeal cancer cases using E2/E6 ratio analysis [35]. Accordingly, a previous study identified a high proportion of purely integrated (48%) and mixed (17%) forms in 23 paraffin-embedded HNSCC cases using E2/E6 ratio analysis [36]. It is significant to highlight that a high frequency of integration events (71%) has been detected in HNSCCs using next generation sequencing [37]. A more recent study revealed that HPV16 DNA was partly or fully integrated in all 20 HPV16-positive laryngeal squamous cell carcinomas using E2/E6 ratio analysis [38]. In contrast, Faust et al. [39] revealed higher rates of mixed (42%) and purely episomal forms (51%) in HNSCC cases, while the purely integrated form was detected in only 6% of the examined HNSCC samples using E2/E6 ratio analysis. As is evident, the vast majority of studies have examined the integration of viral DNA in HNSCC cases using E2/E6 ratio analysis [35,36,37,38,39], providing different frequencies in viral integration events. This discrepancy in research outcomes occurs probably due to differences in the sites of the *E2* gene disruption. Previous analyses concerning cervical dysplasia have proved that the HPV16 *E2* gene is disrupted in various sites, while the distribution of preferential sites of gene disruption varied among different examined populations [15,40,41]. Thus, it has been suggested that an extensive analysis of the *E2* gene should be performed prior to E2/E6 analysis in a given population in order to select the most suitable primer sets targeting the *E2* gene [15]. In the present analysis, the selection of primer sets was conducted considering the most prevalent sites of *E2* gene disruption of HPV16 strains that are circulating in the Greek population in order to obtain more accurate results [41]. It would certainly be interesting to examine whether HPV16 integration frequency in HNSCC cases is associated with the HPV16 strains that are circulating in different populations as well as whether viral integration incidence is related to different HNSCC characteristics, including tumor grade and anatomic position. According to our results, it was demonstrated that the mixed HPV16 DNA form is the most common form (42.1%) among the examined oropharyngeal tumors, followed by purely episomal (31.6%) and purely integrated (26.3%) forms. Considering the high proportion of integrated viral DNA, either in a mixed or purely integrated form, it was postulated that viral integration events might have a considerable impact on oropharyngeal cancer development. Nevertheless, more analyses are required to be conducted in order to estimate whether HPV16 DNA physical status can be utilized as a potential biomarker for the prediction of oropharyngeal cancer development.

In conclusion, in our cohort of Greek patients with HNSCC, we attempted to understand whether viral genes’ epigenetic alterations and different physical status of the virus are related, with the intention of adding important knowledge to understanding the biology of HNSCC in the presence of HPV. Undeniably, further studies on a larger scale are needed to ascertain the exact functional significance and underlying mechanisms of these associations. Such studies will help elucidate the specific regulatory roles of methylation in the UTR and *L1* HPV 16 genes and deepen our understanding of the epigenetic control of viral gene expression and HPV-related HNSCC.

## 4. Materials and Methods

**Figure 2:**
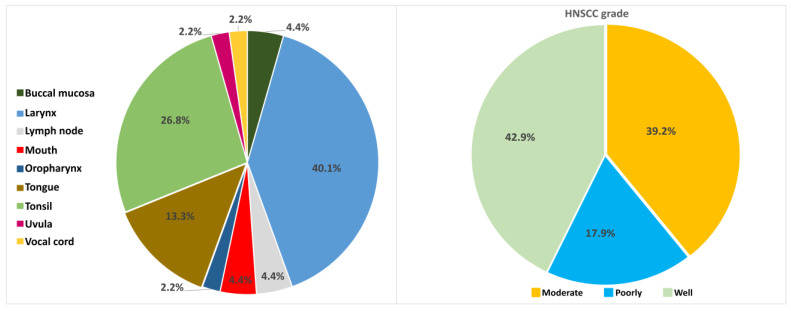
(**Left**): distribution of different anatomical sites where tumor derived; (**Right**): histology grading of studied samples.

Histological sections were obtained from HPV16-positive HNSCC FFPE biopsies of 38 Greek patients aged between 45 and 82 years (median = 57 years). The histology grade and the tumor anatomic position are presented in Figure 2. The HPV genotyping was performed by using a commercially available real-time PCR assay kit (HPV Genotypes 14 Real-TM Quant, Sacace Biotechnologies, Como, Italy) after the DNA extraction with QIA amp DNA FFPE Tissue Kit (Qiagen GmbH) following the manufacturer’s instructions. The extracted DNA concentration was measured with QIAexpert technology (Qiagen, Heidelberg, Germany).

DNA was bisulfite converted with the application of EpiTect Bisulfite Kit (Qiagen, Heidelberg Germany) using the protocol for Sodium Bisulfite Conversion of Unmethylated Cytosines in DNA from Low-Concentration Solutions, following the manufacturer’s instructions and stored at −20 °C. For the quantification of the methylation of CpG sites (PyroMark Q24, Qiagen, Heidelberg, Germany), biotin-labeled primer sets, PCR conditions and sequencing primers were used in validated pyrosequencing assays already published [29,33]. Methylation investigation was conducted for 6UTR CpG sites (31, 37, 43, 52, 58, and 7862) and 6 CpG sites along the *L1* gene (7034, 7091, 7136, 7145, 6367, 6457).

Subsequently, the studied samples were investigated for the physical status of HPV16 DNA. The episomal, mixed and integrated viral DNA forms were determined through E2/E6 DNA copy number ratios using the assay of quantitative Real Time—PCR. The main principle of the methodology is based on the fact that E2 and E6 genes are present in equal amounts in episomal viral DNA. On the other hand, HPV16 DNA integration leads to the disruption of the *E2* gene, while the E6 gene remains intact and integrated into the host chromosome. To this end, two different plasmid constructs were designed as previously reported [15]. In particular, a plasmid containing a partial fragment of the GAPDH gene (pGAPDH) was constructed for the normalization of genomic DNA, whereas a plasmid containing the HPV16 portion from the *E6* to *E2* genes (pE6-E2) was constructed to normalize the RT-PCR assays. The copy numbers of *E2* and *E6* genes were measured considering the number of cells through the quantitative RT-PCR targeting the GAPDH gene. The results were expressed as E2, E6 copy number per 500 cells. Real-Time PCR conditions, primers and cut-off values were performed as previously described [15].

Data were recorded in Microsoft Excel 2016 (version 2308) spreadsheets (Microsoft Corporation, Redmond, Washington, DC, USA), with rows representing cases and columns variables. Statistical analysis was performed using the SAS for Windows, version 9.4 software platform (SAS Institute Inc., Cary, NC, USA). Descriptive values were expressed via the mean value and the standard deviation (SD) and, for completeness reasons, the median value, along with the quartile 1 (Q1) to quartile 3 (Q3) range, was also provided. For the categorical data, the relevant frequencies per category and percentages are provided. Comparisons among the groups of the methylations in the studied sites were performed using non parametric tests, since normality, as tested with the Shapiro Wilk test, was not always ensured. Specifically, the Kruskal–Wallis test was used to compare among more than two categories. In order to investigate for possible correlations between the methylation at the studied sites, the non-parametric Spearman correlation coefficient was used. Correlation coefficients less than 0.20 are characteristic of no correlation, between 0.20 and 0.39 are characterized as weak, between 0.40 and 0.59 as moderate, between 0.60 and 0.79 as strong and >0.80 as very strong. The significance level (*p*-value) for the study was set tο 0.05, and all tests were two-sided.

## Figures and Tables

**Table 1:** Mean methylation and standard deviation for each CpG site studied.

CpG Site	Mean Methylation ± SD
L1 6367	38.16 ± 12.60
L1 6457	37.11 ± 24.43
L1 7034	37.45 ± 24.65
L1 7091	41.47 ± 23.74
L1 7136	43.84 ± 16.69
L1 7145	43.11 ± 17.26
UTR 31	24.74 ± 16.35
UTR 37	31.00 ± 21.45
UTR 43	31.42 ± 23.11
UTR 52	32.55 ± 23.03
UTR 58	28.37 ± 19.70
UTR 7270	12.37 ± 10.46
UTR 7862	9.71 ± 13.85

**Table 2:** Median methylation and Q1-Q3 range according to the histological grade and methylation quantification for each CpG site studied.

CpG Site	Histology Grade			*p*-Value
	Poorly	Moderate	Well	
L1 6367	42 (39–49)	37 (27–47)	45 (40.5–50)	0.2607
L1 6457	23 (19–25)	35 (25–58)	39 (11–61.5)	0.1259
L1 7034	21 (16–51)	44 (35–58)	33 (15–61)	0.4725
L1 7091	40 (35–45)	39 (21–52)	49.5 (25.5–75.5)	0.5901
L1 7136	34 (21–37)	43 (31–47)	57.5 (52–60)	0.0014
L1 7145	29 (20–29)	40 (32–44)	56.5 (52.5–65.5)	0.0015
UTR 31	10 (2–38)	14 (10–32)	32 (22–34)	0.2312
UTR 37	14 (13–41)	18 (11–48)	39 (28–45.5)	0.3768
UTR 43	18 (2–47)	28 (7–47)	38 (32–57)	0.1573
UTR 52	22 (11–47)	16 (12–46)	36 (25.5–51.5)	0.3909
UTR 58	15 (10–46)	15 (9–37)	37.5 (27–41)	0.2920
UTR 7270	17 (8–21)	6 (3–17)	10.5 (3.5–18)	0.6257
UTR 7862	8 (7–10)	8 (5–12)	6.5 (5–8)	0.6219

**Table 3:** Median and Q1-Q3 range for the methylation sites and Spearman correlation coefficients. Values in square brackets indicate 95% confidence intervals. ***: *p* < 0.0001, **: *p* < 0.001, *: *p* < 0.05.

	Median (Q1–Q3)	UTR_37	UTR_43	UTR_52	UTR_58	UTR_7862	UTR_7270s	L1_7034	L1_7091	L1_7136	L1_7145	L1_6367	L1_6457
UTR_31	27 (11–35)	0.92 [0.85–0.95] ***	0.94 [0.89–0.96] ***	0.93 [0.88–0.96] ***	0.92 [0.85–0.95] ***	0.3 [0–0.54] *	0.12 [−0.18–0.4]	0.13 [−0.18–0.4]	0.31 [0.01–0.55] *	0.59 [0.35–0.75] ***	0.63 [0.41–0.78] ***	0.38 [0.09–0.6] *	0.49 [0.22–0.68] **
UTR_37	31 (13–45)		0.91 [0.84–0.95] ***	0.94 [0.89–0.97] ***	0.92 [0.85–0.95] ***	0.27 [−0.03–0.52]	0.14 [−0.16–0.41]	0.11 [−0.19–0.39]	0.3 [0.01–0.55] *	0.56 [0.32–0.73] ***	0.59 [0.35–0.75] ***	0.35 [0.05–0.58] *	0.47 [0.19–0.67] *
UTR_43	32 (10–47)			0.92 [0.86–0.96] ***	0.92 [0.85–0.95] ***	0.29 [−0.01–0.53]	0.08 [−0.22–0.36]	0.09 [−0.21–0.37]	0.33 [0.03–0.56] *	0.58 [0.34–0.74] ***	0.65 [0.43–0.79] ***	0.41 [0.13–0.62] *	0.47 [0.19–0.67] *
UTR_52	32 (13–47)				0.91 [0.83–0.95] ***	0.31 [0.02–0.55] *	0.14 [−0.16–0.42]	0.11 [−0.19–0.39]	0.35 [0.05–0.58] *	0.59 [0.35–0.75] ***	0.63 [0.4–0.77] ***	0.4 [0.11–0.62] *	0.42 [0.14–0.64] *
UTR_58	24 (13–41)					0.27 [−0.03–0.52]	0.1 [−0.2–0.38]	0.1 [−0.2–0.38]	0.33 [0.03–0.56] *	0.51 [0.26–0.7] **	0.59 [0.35–0.75] ***	0.42 [0.14–0.63] *	0.4 [0.12–0.62] *
UTR_7862	7 (5–10)						0.19 [−0.11–0.46]	−0.03 [−0.32–0.27]	−0.1 [−0.38–0.2]	0.17 [−0.13–0.44]	0.11 [−0.19–0.39]	0.16 [−0.14–0.43]	0.12 [−0.19–0.4]
UTR_7270	11 (4–21)							0.15 [−0.15–0.43]	0.16 [−0.14–0.43]	0.4 [0.11–0.61] *	0.31 [0.01–0.55] *	0.26 [−0.04–0.51]	0.12 [−0.18–0.4]
L1_7034	35 (16–54)								0.51 [0.25–0.69] **	0.26 [−0.04–0.51]	0.19 [−0.11–0.46]	0.3 [0–0.54] *	0.07 [−0.24–0.35]
L1_7091	41 (24–53)									0.39 [0.1–0.61] *	0.43 [0.15–0.64] *	0.48 [0.21–0.68] **	0.09 [−0.22–0.37]
L1_7136	47 (34–56)										0.9 [0.83–0.94] ***	0.46 [0.19–0.66] *	0.37 [0.07–0.59] *
L1_7145	44 (29–53)											0.46 [0.19–0.66] *	0.41 [0.12–0.62] *
L1_6367	42 (35–49)												0.17 [−0.13–0.45]
L1_6457	27.5 (16–52.5)												1

## Data Availability

Not applicable.
